# Excitation of dark multipolar plasmonic resonances at terahertz frequencies

**DOI:** 10.1038/srep22027

**Published:** 2016-02-23

**Authors:** Lin Chen, YuMing Wei, XiaoFei Zang, YiMing Zhu, SongLin Zhuang

**Affiliations:** 1 Shanghai Key Lab of Modern Optical System, and Engineering Research Center of Optical Instrument and System(Ministry of Education), University of Shanghai for Science and Technology, No. 516 JunGong Road, Shanghai 200093, China; 2 Cooperative Innovation Center of Terahertz Science, Chengdu 611731, China

## Abstract

We experimentally observe the excitation of dark multipolar spoof localized surface plasmon resonances in a hybrid structure consisting of a corrugated metallic disk coupled with a C-shaped dipole resonator. The uncoupled corrugated metallic disk only supports a dipolar resonance in the transmission spectrum due to perfect symmetry of the structure. However, the dark multipolar spoof localized surface plasmon resonances emerge when coupled with a bright C-shaped resonator which is placed in the vicinity of the corrugated metallic disk. These excited multipolar resonances show minimum influence on the coupling distance between the C-shaped resonator and corrugated metallic disk. The resonance frequencies of the radiative modes are controlled by varying the angle of the C-shaped resonator and the inner disk radius, both of which play dominant roles in the excitation of the spoof localized surface plasmons. Observation of such a transition from the dark to radiative nature of multipolar spoof localized plasmon resonances would find potential applications in terahertz based resonant plasmonic and metamaterial devices.

Surface plasmon polaritons (SPPs) are the electromagnetic waves propagating at the flat interface between a conductor and a dielectric. They are coupled to an electric charge density fluctuation on the metallic surface^1^. The propagation constant is greater than the wave vector in the dielectric, so that the wave is confined leading to evanescent decay on both sides of the interface. Because the SPPs are usually limited to the sub-wavelength range, they could achieve subwavelength spatial resolution and the strong field enhancement effect, which have widespread applications in near-field optics^2^, Plasmon antennas^3^, and surface-enhanced phase shift^4^. Besides SPPs on the flat metal surface, localized surface plasmons (LSPs) arise around the metallic nanoparticles or nanoshells^5^,^6^,^7^. LSPs originate from the scattering effect of a sub-wavelength conductive nanoparticle in an oscillating electromagnetic field. The closed curved surface of the metallic particle exerts an effective restoring force on the driven electrons, leading to field amplification both inside and in the near-field zone outside the particle. Thus, different from SPPs, LSPs are non-propagating excitations of the conduction electrons in metallic nanostructures coupled to the electromagnetic field. In the visible and near-infrared regions, the frequency is much greater than the collision frequency of the metal (on the order of 10^14^ Hz), resulting in the coupling of the electromagnetic field to the free charges in the metal and the strong interaction strength between the wave and electron plasma with small damped influence. At the very low frequencies of the microwave and terahertz regions, however, the frequency is much lower than the collision frequency. The driving field comes from bound charges and dissipation decreases clearly due to the fact that the depth of the wave penetration into the metal is much shorter than the corresponding wavelength (the skin depth is on the order of 100 nm), leading to the weak interaction between the wave and electron plasma at these frequencies^8^,^9^,^10^,^11^. Then, instead of metallic particle, a periodic textured metallic disk structure was theoretically proposed to support spoof LSPs^12^. Such a designed metallic curved surface with subwavelength grooves can achieve the confinement of longer wavelength electromagnetic waves. Thus, localized surface plasmon-like modes are supported with the electric field localized more strongly on the metallic particle surface. These novel spoof LSPs had been experimentally verified in the microwave regime^13^,^14^,^15^,^16^,^17^. At terahertz frequencies, only the dipole LSP was successfully excited and studied^9^,^18^. However, the experimental verification of multipolar spoof LSPs has not been reported in the terahertz range because the excitation of the coaxial antenna (or monopole) source cannot extend its band into the terahertz range in the case of grazing incidence. Therefore, novel designs and structures that could excite terahertz multipolar spoof LSPs still require a thorough investigation.

In this contribution, we theoretically and experimentally demonstrate the excitation of terahertz multipolar spoof LSP resonances in the transmission spectra at normal incidence in a hybrid structure consisting of a corrugated metallic disk (CMD) coupled with a C-shaped resonator (CSR). One may ask why multipolar spoof LSPs could not be observed experimentally in terahertz transmission spectra until now. The bottleneck is that this multipolar spoof LSPs in CMD can be seen as dark modes which cannot be directly excited by normal incident wave in symmetric structures. Here we added a “bright” CSR in the vicinity of CMD and experimentally observed the evident transmission dips corresponding to resonant modes of high azimuthal order (dipole to decapole modes). The physical origin of this phenomenon is due to strong coupling between the bright resonant mode of parallel CSR (the tangent at the center of CSR is parallel to the polarized direction of the incident wave) and the dark multipolar modes of CMD^19^,^20^. In addition, the unique features of this near-field coupling are discussed by changing three parameters: the gap between CSR and CMD, the angle of CSR, and the inner disk radius. The property of a hybrid structure with asymmetric CMD is also investigated. Moreover, we found that the quadrupole and octupole modes does not exist if such hybrid structure is changed by placing second identical CSR symmetrically with respect to the first one. Both hybrid structures show polarization dependence. The observed results in this work provide a new perspective to engineer terahertz multipolar spoof LSPs which could be extremely useful in the next generation active and passive components, such as sensors, filters and modulators.

## Results

### Localized surface plasmons in corrugated metallic disk

**Figure 1:**
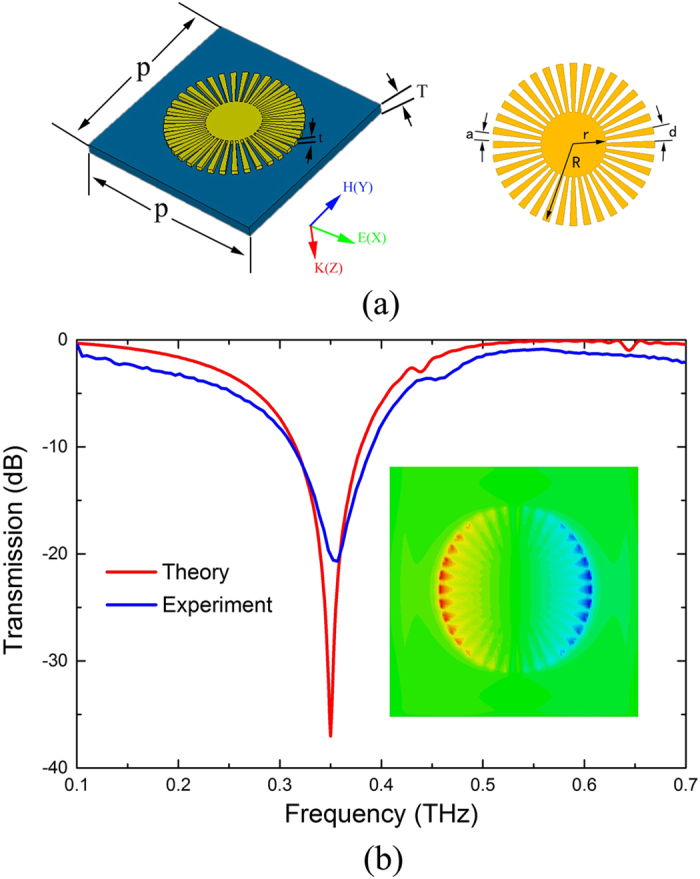
(**a**) The geometric parameters of the CMD. (**b**) Transmission spectra of the symmetric CMD. The inset shows electric field (*E*_*z*_) distribution at 0.35 THz resonance, which can be verified as dipole mode.

First, we analyzed the transmission spectra at the normal incident on a CMD at terahertz frequencies. In Fig. 1(a), we schematically illustrate the geometry of CMD, which is composed of outer disk radius *R* = 150 *μ*m and inner metallic disk of radius *r* = 60 *μ*m surrounded by total of *N* = 36 grooves with the periodicity *d* = 2π*R*/*N*. The parameter *α* = *a/d* = 0.4 is the air-filled ratio in the single periodic structure (*a*: groove width) and thickness of the metallic film (aluminum, *σ*_Al_ = 3.56 × 10^7^ S.m^−1^) disk *t* = 200 nm.The metallic disk is based on a 25 *μ*m-thick polyimide substrate with dielectric constant of 3.5 and loss tangent of 0.05^21^. The period of the unit cell is *p* = 420 *μ*m. From the experiment point of view, it is technically difficult to verify these spoof LSP modes in grazing incidence at terahertz frequencies due to the lack of suitable terahertz sources and lower side coupling coefficient. Here, we fabricated the sample using conventional photolithography (the geometric parameters are chosen to be same as in Fig. 1(a)) and measured the transmission spectrum of this sample by using fast and slow scan-based terahertz time-domain spectroscopy (THz-TDS) systems^21^,^22^,^23^,^24^, as shown in Fig. 1(b) (the simulated results are also shown for comparison). The resonance frequency for simulation (experiment) is 0.35 THz (0.357 THz) and the Q-value of resonance for simulation (experiment) is 14 (4.6).There is only one resonance observed experimentally. The electric vertical (*E*_*z*_) distribution on the plane 2 *μ*m above the configuration at this resonance frequency (0.35 THz) in the inset of Fig. 1(b) verified that it is indeed a dipole mode. As a result, in this symmetric CMD, the normal incident wave cannot propagate along the groove and excite higher order azimuthal standing surface waves.

### Spoof localized surface plasmons in corrugated metallic disk coupled to a C shaped dipole resonator

**Figure 2:**
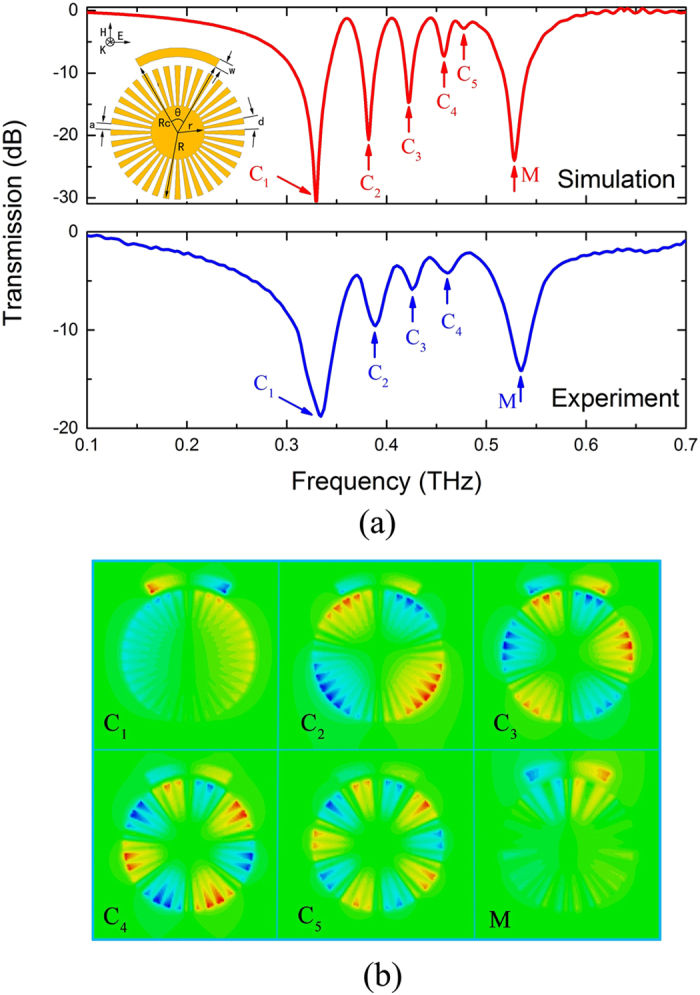
(**a**) Theoretical (top) and experimental (bottom) transmission spectra of the proposed hybrid structure. The inset shows the schematic diagram of the CMD and CSR hybrid structure. The angle of CSR *θ*, the CSR inner radius *R*_c_ and width *w* of CSR are also marked (**b**) *E*_*z*_ field distribution of multipolar resonance frequencies at 0.329 THz (C1, dipole), 0.382 THz (C2, quadrupole), 0.422 THz (C3, hexapole), 0.458 THz (C4, octupole), 0.477 THz (C5, decapole), and 0.528 THz (M). The resonance (M) comes from the bright LSP mode supported by the single parallel CSR structure.

Figure 1 indicates that only the main dipole mode can be directly excited by normal incidence in CMD. To excite multipolar resonances at normal incidence by using THz-TDS, we introduce asymmetry in the CMD structure. In this section, we proposed the hybrid spoof LSP structure consisting of one CMD and one CSR structure. The inset of Fig. 2(a) depicts the schematic diagram of the CMD and CSR hybrid structure. The angle of CSR *θ* (60^o^), the CSR inner radius *R*_c_ (170 *μ*m) and width *w* (20 *μ*m) of CSR are also marked in the inset of Fig. 2(a). The other parameters are the same as Fig. 1(a). Then the gap between CMD and CSR is *g* = *R*_c_ – *R* = 20 *μ*m. The terahertz wave with electric field parallel to the CSR arc illuminates the sample at normal incidence.

This hybrid structure can excite spoof LSP modes. To see this clearly, Fig. 2(a) shows the simulated and the experimental transmission spectra of the proposed structure that consists of one CMD and one CSR. We focused on the spoof LSPs spectrum band (0.1–0.7 THz). Apparent multipolar resonances (marked by C1–C5) could be found theoretically and most of these resonances (C1–C4) were observed experimentally. The higher resonance (marked by M) arises from the bright LSPs excited by the CSR. To investigate the underlying physics of the multipolar resonances, the electric field *E*_*z*_ corresponding to dips C1–C5 and M are shown in Fig. 2(b). The CSR is resonant as a LSP mode M, while the disk shows multipolar modes, which are in accordance with the spoof LSP modes of a CMD. These five resonant modes correspond to di- (C1), quadru- (C2), hexa- (C3), octu- (C4) and decapolar (C5) resonance modes. This phenomenon is similar to that observed at the grazing incidence in the CMD structure^13^. By investigating the field response (Fig. 2(b)), we can find that the CSR is directly dipole excited by the incident parallel electric field along the arm. There is direct electrical dipole coupling with the radiation wave. Then the near field electric coupling between the CSR and the CMD excites the multipolar resonances in the CMD. At this time the CSR and the CMD are both excited. Excitation of the dark CMD couples back to the bright CSR, forming a high electric field at its end facets. The electric field is coupled back and forth between the bright LSP resonance and dark multipolar resonances. So the coupling mechanism is coupled electric-dipoles response in the metallic metamaterials, and the CMD is excited by the local electric field from electric resonance of CSR. As a result, the resonance M mainly originates from the bright resonant mode of parallel CSR, while the dark multipolar modes from CMD cannot be directly excited to resonate at normal incidence. The images in Fig. 2(b) prove that the multipolar modes of the CMD are excited by the bright LSP mode of CSR. In our system, there is only one dark CMD and the dark multiple resonances are induced to be resonant as multipolar modes with spoof LSPs. The resonance frequencies of modes C1–C4 and M for simulation (experiment) are 0.329 THz (0.334 THz), 0.382 THz (0.389 THz), 0.422 THz (0.426 THz), 0.458 THz (0.462 THz) and 0.528 THz (0.535 THz), respectively. There are little deviations between the simulation and measurement results. It is also noted that the Q-values of resonances marked as C1, C2, C3 and C4 for simulation (experiment) are 18.3 (9.6), 29.4 (21.2), 38.4 (29.8) and 50.9(33.7), respectively, which are all higher than the simulation (experiment) Q-value 14(4.6) of dipole resonance in Fig. 1(b). The higher the azimuthal order resonance has, the higher the Q-value. Numerical results show good agreement with experimental results.

**Figure 3:**
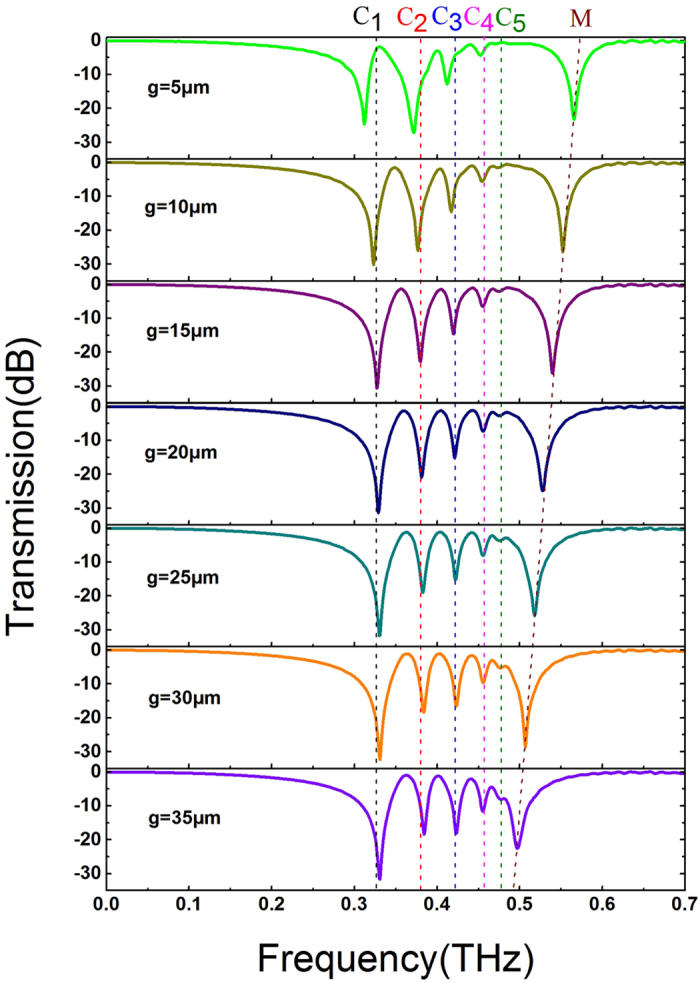
Transmission spectra with respect to various gaps *g* between CSR and CMD. With the increase of gap *g*, the resonance frequencies of C1–C5 have blueshifted at the beginning and maintain constant soon afterwards.

To further reveal the field distribution of excited multipolar spoof LSPs, we have studied how the spoof LSPs resonances are affected by varying the gap ‘*g*’ between CMD and CSR, as shown in Fig. 3.With *g* increased, the dips C1–C5 slightly blueshift. The electric distribution of CMD and CSR are out of phase at dips C1–C5 (see Fig. 2(b)). When *g* becomes larger, the coupling strength between the CMD and CSR is decreased, so the attraction between the CSR and CMD for multipolar modes is decreased. On the contrary, the dip M has redshifted with increase in the gap *g*. This is because the electric field is in phase for the CMD and CSR at dip M (see Fig. 2(b)), and the repulsion effect in the hybrid structure is decreased when *g* is increased. Therefore the dip M redshifts. As a result, the resonance frequencies of spoof LSPs have slightly blue shifted by increasing gap *g*, as shown in Fig. 3.

**Figure 4:**
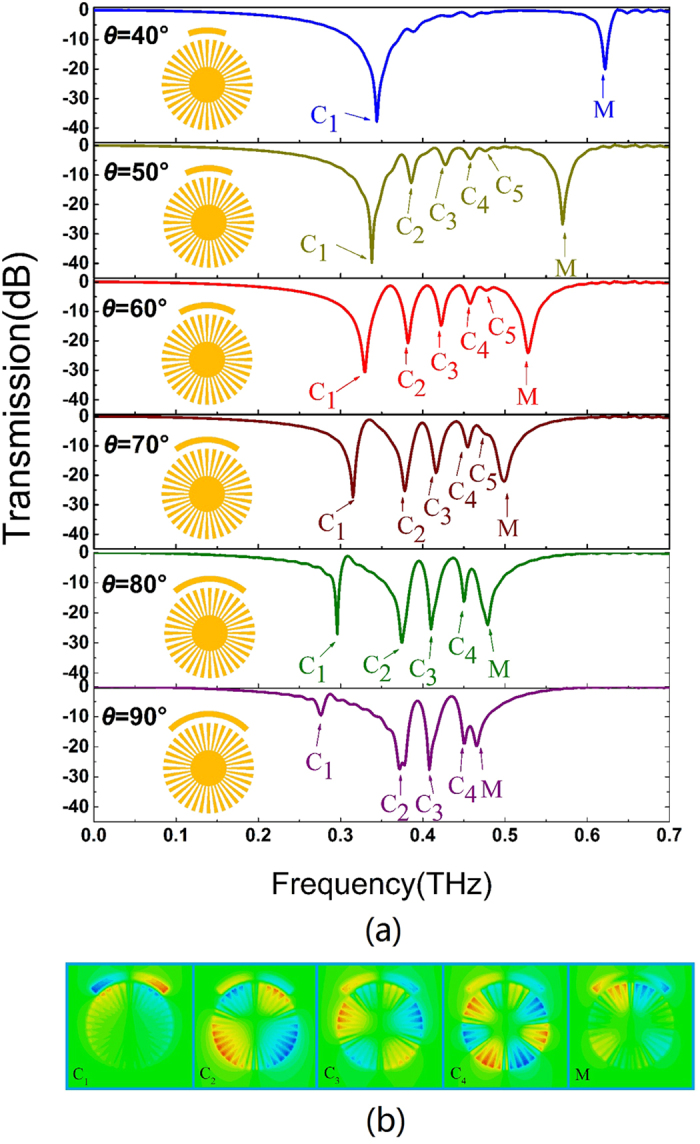
(**a**) Transmission spectra with respect to various angles of CSR. This angle can determine the resonance frequency of CSR and further affect the excitation of spoof LSPs. (**b**) *E*_*z*_ field distribution of multipolar resonance frequencies for *θ* = 90°.

The resonance frequency of CSR can be tuned by the angle *θ* of CSR, which can further influence the intensity and position of multipolar spoof LSPs in the transmission spectra. To see this clearly, the angle of CSR is increased from 40^o^ to 90^o^ and the gap *g* is fixed to 20 *μ*m. The transmission spectra of the hybrid structure are plotted in Fig. 4(a) at different angles. When *θ* = 40^o^, only dipole mode C_1_ can be seen in the transmission spectrum. This is because the mode of CSR is resonant at 0.64 THz, which is far away from the resonance frequency of nearest multipole (decapole). So it cannot successfully excite the dark multipolar spoof LSPs of CMD, resulting in only dipole mode similar to Fig. 1(b). As *θ* is increased, the resonance frequency of CSR redshifts and is gradually close to the decapolar resonance frequency. The quadru-, hexa-, octu- and decapolar modes are excited successively and enhanced. When *θ* is further increased above 70^o^, the resonance frequency of CSR is gradually close to the high order spoof LSP resonance (*θ* = 80^o^) and then overlapped with it (*θ* = 90^o^). We also investigate *E*_*z*_ field distribution of all resonance frequencies for *θ* = 90^o^, as in Fig. 4(b). The angle of CSR *θ* has a significant impact on the dipole resonance (C1). This is because the length of CSR according to *θ* plays a significant role to asymmetry of the structure along X axis. The larger the length of CSR is, the more serious the asymmetry along X axis is, and the weaker the dipole resonance is, as shown in C1 image in Fig. 4(b). In addition, the CSR resonance (M) is weakened as it overlaps the octupole resonance (C4) for *θ* = 90^o^. The M image in Fig. 4(b) indicates that such CSR resonance has gradually evolved into another octupole resonance with the opposite phase of C4. As a result, the existence of CSR not only excites spoof LSPs, but also has directly strong interaction and interference with spoof LSPs, resulting in more complex resonances in the transmission spectrum.

**Figure 5:**
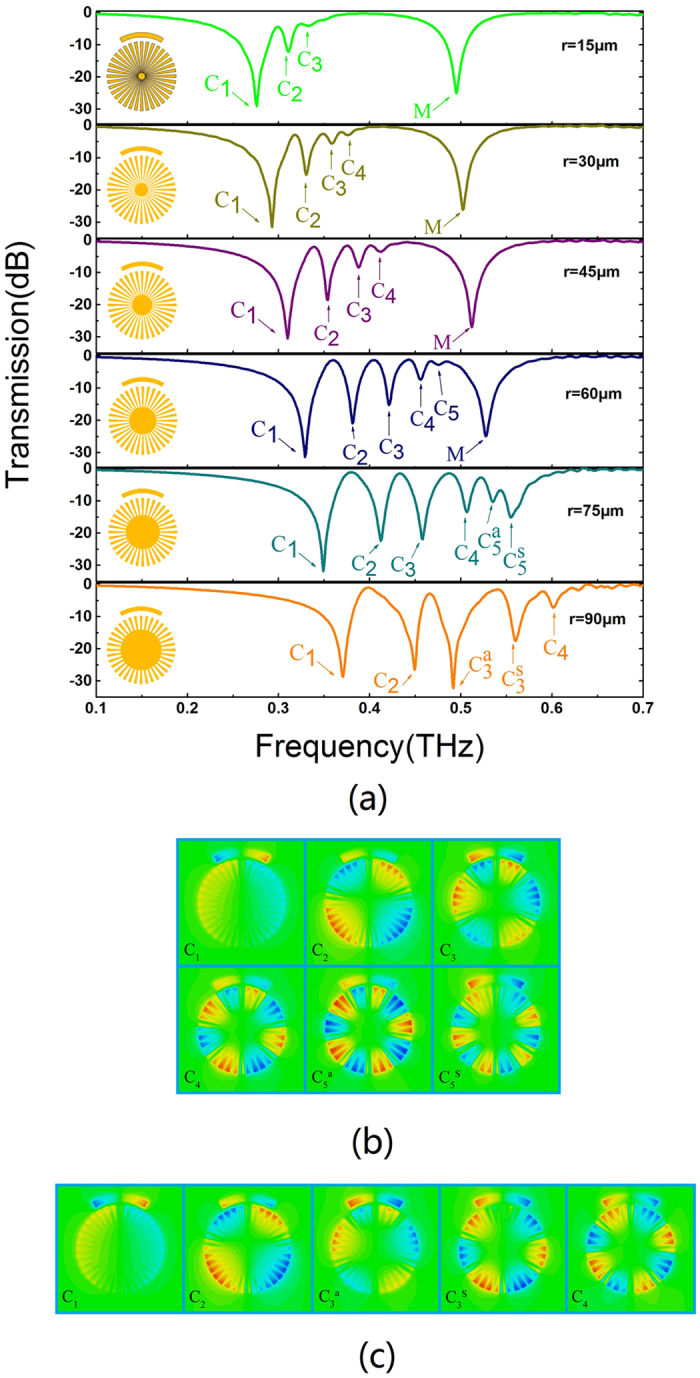
(**a**) Transmission spectra with respect to various inner disk radius r. (**b**) *E*_*z*_ field distribution of multipolar resonance frequencies for *r* = 75 *μ*m; (**c**) *E*_*z*_ field distribution of multipolar resonance frequencies for *r* = 90 *μ*m.

Next, we analyze the influence of transmission spectra on the CMD inner radius *r*. The simulated dispersion curves of the corresponding corrugated metallic strips are shown in Supplementary Information Fig. S2 with *R* = 150 *μ*m, *N* = 36, *d* = 2π*R*/*N*, *a* = 0.4*d* and different inner radius *r*. The transmission spectra with different *r* are plotted in Fig. 5(a). It is clearly observed that the blueshift property of LSP resonance frequencies with inner radius *r* plotted in Fig. 5(a) is actually consistent with the dispersion relations shown in Supplementary Information Fig. S2. When the inner radius *r* increases from 15 *μ*m to 90 *μ*m, the asymptote frequency of dispersion curves increases from 0.35 THz to 0.752 THz, and blueshifts of the spoof LSPs frequencies are observed(Fig. 5(a)). We note that the highest order resonances of spoof LSPs (in Fig. 5(a)) approximate the corresponding asymptote frequencies (in Supplementary Information Fig. S2). The slight variance is owing to the difference of radial widths of grooves in spoof SPP and LSP. As spoof LSP resonance frequency is close to asymptote frequency, the propagation of terahertz wave is slower and its field is more tightly confined to the corrugated metal disk, leading to weakening of the intensity of the corresponding spoof LSPs, as shown in Fig. 5(a). In addition, for *r* = 75 *μ*m and 90 *μ*m, the asymptote frequencies are 0.612 THz and 0.752 THz, respectively, which overlap the resonance frequency of CSR (in Fig. 5(a)). The electric fields of resonance frequencies for *r* = 75 *μ*m (in Fig. 5(b)) and 90 *μ*m (in Fig. 5(c)) show that some higher order spoof LSP modes exhibit mode splitting effect, which may be due to the interaction between spoof LSP mode and CSR mode. We also fabricated the sample for *r* = 75 *μ*m and experimental transmission spectrum agrees well with the simulation results, as shown in Supplementary Information Fig. S3. As a consequence, the inner radius *r* has a significant impact on the spoof LSP resonance frequency. In this work we choose *r* = 60 *μ*m to support the highest order radial spoof LSP (decapole). Moreover, the asymptote frequency and related spoof LSP resonance frequency are nearly independent of the filling ratio *α*(*a/d*) and numbers of sectors *N*. Detailed accounts of this phenomenon are discussed in depth in the Supplementary Information.

### Spoof localized surface plasmons supported by hybrid structure consisting of two identical CSR placed symmetrically on top and bottom of the CMD

**Figure 6:**
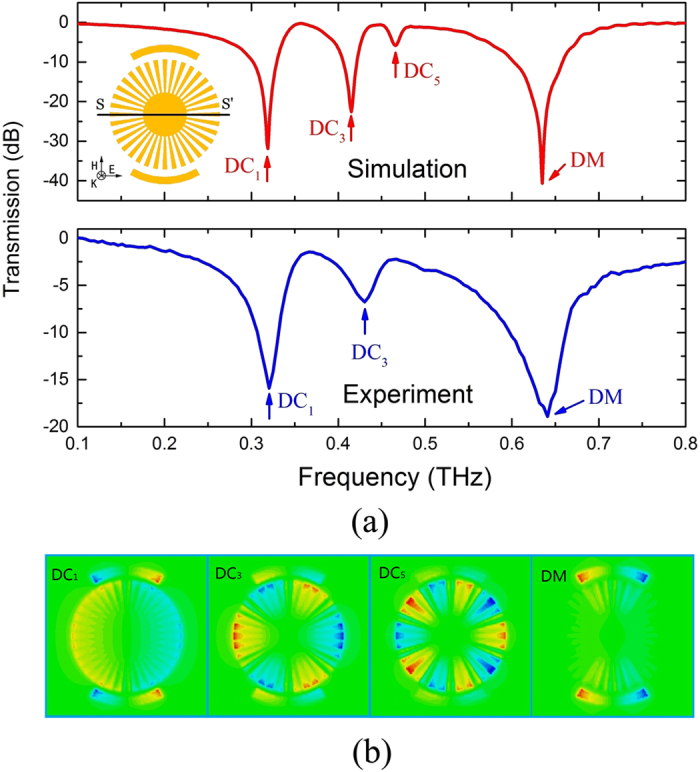
(**a**) Theoretical(top) and experimental(bottom) transmission spectra of the hybrid structure consisting of two CSRs and one CMD. Another identical CSR is placed symmetrically with respect to the first one, equidistant from the CMD center. (**b**) *E*_*z*_ field at resonance frequencies 0.319 THz (DC1, dipole), 0.415 THz (DC3, hexapole), 0.466 THz (DC5, decapole), and 0.635 THz (DM).

To further investigate the unique feature of spoof LSPs in the terahertz range, we fabricate and discuss a hybrid structure consisting of two CSRs and one CMD in this section. Figure 6(a) shows the theoretical and experimental transmission spectra of this hybrid structure where another identical CSR is added at the opposite side of CMD symmetrically and all the other parameters are the same as those of the structure in Fig. 2. The inset of Fig. 6(a) (top) depicts the schematic configuration of the proposed hybrid structure. Four resonances marked as DC1, DC3, DC5 and DM could be seen in the simulation spectrum. The experimental result fits well with theory except DC5, which may be due to additional loss in the sample. The resonance frequencies of modes DC1, DC3, and DM for simulation (experiment) are 0.319 THz (0.32 THz), 0.415 THz(0.43 THz), and 0.635 THz(0.64 THz), respectively. The Q-values of DC1 and DC3 for simulation (experiment) are 24.5 (10), and 30.7 (13.4), respectively. The electric fields at resonance frequencies of DC1, DC3, DC5 and DM are also shown in Fig. 6(b). Only multipolar spoof LSP modes of dipole (DC1), hexapole (DC3) and decapole (DC5) can be supported in this structure. The reason is as follows: the structure is symmetrical along the SS’ axis(see the inset of Fig. 6(a)), so the phase of the electric field in CMD has symmetric distribution along SS’ axis. The modes (quadrupole, octupole, etc) could not satisfy this condition, thus cannot be excited in the transmission spectrum. Here we note that the simulated resonance frequencies of dipole, hexapole and decapole in Fig. 6(a) are close to those with the same order in Fig. 2.

**Figure 7:**
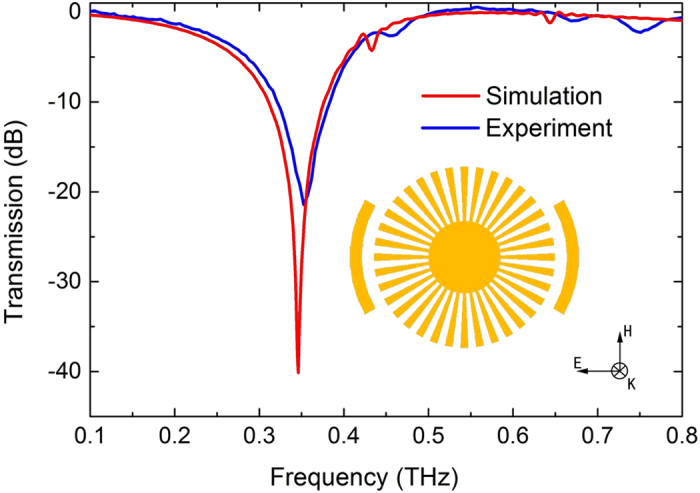
Simulated and measured transmission spectra with the polarization direction of 90^o^ for the structure shown in Fig. 6. The resonance frequency for simulation (experiment) is 0.346 THz (0.352THz). The Q-value of resonance for simulation (experiment) is 20.4 (6.4).

Next, we examine the dependence of the transmission spectra on the polarization of the incidence. We assume that the polarization direction along the X (or SS’) axis is 0°, and rotate the sample by 90° in the XOY plane to change the polarization direction with 90°. The simulation and measurement results of the hybrid structure with two perpendicular CSRs and one CMD are illustrated in Fig. 7. Only dipole mode can be found which is similar to the results shown in Fig. 1. As we know, for the electric-field vector parallel to the X (or SS’) axis (0°), the incident wave polarization and the tangent vector of the CSR arc at the central point have the same direction, and then the bright LSP mode of CSR can be excited efficiently and the interaction between the bright and dark modes becomes the strongest. When the electric-field vector is along with the Y axis (90°), the multipolar resonances disappear and only the dipole mode remains excited for the dark perpendicular CSRs. In this case, there are two dark particles (CSRs and CMD) which should not interact and couple with each other, owing to the fact that the multipolar spoof LSP resonances are diminished. This phenomenon can also be found in the hybrid structure consisting of one CSR and one CMD.

### Localized surface plasmons supported by a hybrid structure consisting of a CSR and an asymmetric CMD

**Figure 8:**
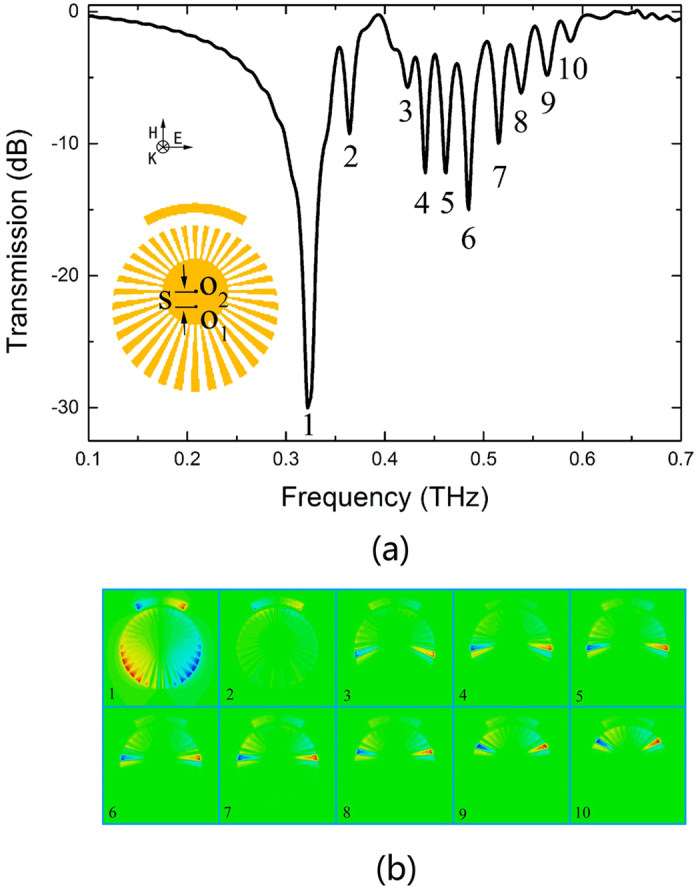
(**a**) Transmission spectrum of the hybrid structure with asymmetric CMD. (**b**) *E*_*z*_ field distribution of resonance frequencies at 0.324 THz (mode 1), 0.364 THz (mode 2), 0.423 THz (mode 3), 0.441 THz (mode 4), 0.462 THz (mode 5), 0.485 THz (mode 6), 0.516 THz (mode 7), 0.538 THz (mode 8), 0.565 THz (mode 9), and 0.589 THz (mode 10).

**Figure 9:**
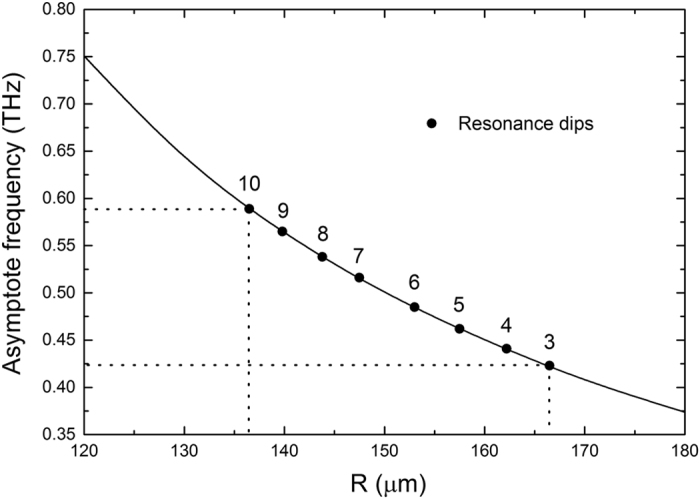
Critical outer radius *R* versus asymptote frequency (or cutoff frequency). Critical radius for each resonance frequency (labeled as 3–10) is marked as dot on the curve. Two dotted lines are also marked to explicitly know the range of *R* with corresponding excited modes.

Here we consider a hybrid structure with graded CMD, as illustrated by the inset of Fig. 8(a). The original inner radius center O_1_ is shifted to O_2_ with 30 *μm* (S) along the positive H(Y) axis. The other parameters (*θ, w, g, r, α*) are the same as Fig. 2. Then the outer radius *R*(the groove depth *h*) changes along the radial propagation direction, from 120(60) *μ*m to 180(120) *μ*m. Figure 8(a) demonstrates transmission spectrum of the hybrid structure with asymmetric CMD. Besides the dominant dipole resonance (marked as 1), there are several other resonances in the spectrum (marked as 2–10), which is different from the structure with symmetrical CMD (Fig. 2(a)). To distinguish these resonances, *E*_*z*_ field distributions of resonance frequencies appeared in Fig. 8(a) are shown in Fig. 8(b). It can be seen that resonance 1(2) shows the strong dipole (weak quadrupole) resonance. However images corresponding to resonances 3–10 indicate that the electrical fields are confined within subwavelength regions along the grating surface, and are localized at different positions along the radial propagating direction^25^. This is because the graded metallic grating has dramatically modified the dispersions of the terahertz wave. The asymptote (cutoff) frequency of the spoof surface plasmons is mainly controlled by the groove depth *h* (*R-r*). In order to gain a deeper insight into their relationship, we plotted the relationship between critical outer radius *R* and asymptote frequency with the fixed *r* = 60 *μ*m, as shown in Fig. 9. Larger *R* (or deeper *h*) corresponds to lower asymptote frequency. When spoof LSPs of different frequencies propagate along the radial surface, they should be localized at certain positions determined by Fig. 9. At this time, the specific spoof LSP resonance frequency is close to the asymptote frequency, the group velocity is reduced to approximately zero, and the terahertz wave is trapped at this location. The results of Fig. 9 are confirmed by *E*_*z*_ field distribution results, shown in Fig. 8(b). These figures indicate that terahertz spoof LSPs can also be trapped in graded CMD at different locations of the surface grating corresponding to the different wavelengths of the waves. In the future work we will analyze and discuss this problem in detail.

## Discussions

It is worth comparing the excitation of dark multipolar plasmonic resonances presented in this work with the known electromagnetically induced transparency (EIT) system^26^,^27^,^28^,^29^,^30^. Their excitation and interaction mechanisms have some common points. For example, a simple metal strip (cut wire, or C shaped arm) can function as a dipole antenna, which shows a typical LSP resonance and serves as the radiative or bright resonator with electric field parallel to the strip in both plasmonic systems; the resonance frequency of such bright resonator can be readily tuned by varying its spatial dimension; and a bright resonator can strongly couple with the incident field while a dark plasmonic atom only weakly couples to the incident wave. However, the difference between them is also obvious. In the plasmonic EIT system, it is necessary to create a very large loss contrast between the dark and bright resonators by many methods (for example, different topologies^26^,^27^, dielectric ceramics with different permittivity loss^28^, employing a superconducting Nb film in the dark element^29^, etc.). So it is convenient to achieve EIT behavior when the bright mode has a low quality factor and the dark mode has a significantly larger quality factor. On the contrary, in our system the dark CMD shows multipolar resonances which do not overlap with the bright mode. The dark multipolar resonances can be efficiently excited only as the bright CSR resonance is close to the asymptote frequency of the CMD. Our work is an important extension and supplement to the coupling and excitation mechanism in EIT system.

In summary, we proposed a hybrid structure consisting of the CMD and CSR on which we demonstrate the excitation of dark multipolar terahertz spoof LSPs at normal incidence. The parallel CSR acts as the bright state and excites the dark multipolar resonances. We experimentally observed the multiple resonances in the transmission spectra and verified their electric distributions that have the nature of dipolar, quadrupolar, hexapolar and octupolar modes in the hybrid structures. If another identical CSR is placed symmetrically with respect to the first one, the new hybrid structure does not support quadrupolar and octupolar modes. The bright resonant mode of CSR can only approach the strongest excitation with the polarization parallel to the CSR length, resulting in polarization dependence in such coupled structures. It is noted that all the structures are fabricated on the flexible material. Since the spoof LSP modes have features of high quality factor and local field enhancement, our proposed structures may have potential applications in multi-channel biosensing and next generation terahertz plasmonic metamaterial devices.

## Methods

### Simulation

The numerical simulations are calculated by using a commercial electromagnetic simulator of CST Studio Suite^®^ 2012. The calculation of dispersion relations of the CMD is based on CST eigenmode solver, where only one unit cell is analyzed and periodic boundary conditions are used. We use transient solver to acquire transmission spectra and electric-field distributions, which is based on the finite-integral time-domain method. Open boundary condition was adopted in the z direction. The electric vertical (*E*_z_) distributions were obtained on the plane 2 *μm* above the configurations at certain frequencies. The polyimide substrate was modeled as a dielectric with *ε*_PI_ = 3.5 and loss tangent 0.05, and Al was simulated with a default conductivity of *σ*_Al_ = 3.56 × 10^7^ S.m^−1^.

### Fabrication and Experimental setup

The spoof LSPs structures were fabricated on the 25*μ*m-thick polyimide(Dupont)by using the traditional photolithography. AZ1500 image reversal photoresist layer with thickness of 1μm was spin-coated and patterned on the polyimide substrate based on the standard photolithography. AZ 300MIF was selected as photographic developer. Then, 200 nm Al film was coated on the substrate by sputter machine, and acetone was used to wash away the remaining part of the photoresist. Finally, the structure was formed on the substrate. The optical microscope and SEM images of the samples are shown in Supplementary Information Fig. S1.

We use the combined fast and slow scan based THz-TDS to measure the transmission spectra. A femtosecond fiber laser was applied to pump and detect terahertz wave, with the central wavelength at 780 nm, output power 150 mW, pulse duration around 90 *f*s, and repeat frequency at 80 MHz. The laser was split into pump and probe beam by a beam splitter. We combine the fast scan with the slow scan to expand the overall delay line to 218.4 *ps*, which can increase the frequency resolution eventually to 4.58 GHz.After passing through slow delay line and fast delay line, the pump beam was introduced into the LTG-GaAs based photoconductive switch (terahertz emitter). The radiated terahertz beam with waist radius ~5 mm was focused to the sample by two off-axis parabolic mirrors. The transmitted terahertz wave from the sample was detected by another LTG-GaAs based photoconductive switch (terahertz detector). The probe beam, which irradiated several components (attenuators, mirrors, lens), was focused on the terahertz detector in order to compensate pump beam path.

## Additional Information

**How to cite this article**: Chen, L. *et al.* Excitation of dark multipolar plasmonic resonances at terahertz frequencies. *Sci. Rep.* **6**, 22027; doi: 10.1038/srep22027 (2016).

## Supplementary Material

Supplementary Information
