# Corrigendum: Excitation of dark multipolar plasmonic resonances at terahertz frequencies

**DOI:** 10.1038/srep27324

**Published:** 2016-06-10

**Authors:** Lin Chen, YuMing Wei, XiaoFei Zang, YiMing Zhu, SongLin Zhuang

Scientific Reports 6: Article number: 22027; 10.1038/srep22027 published online: 02232016; updated: 06102016

In the original version of this Article, an additional affiliation for Lin Chen and YiMing Zhu was omitted. The correct affiliation is listed below:

Cooperative Innovation Center of Terahertz Science, Chengdu 611731, China.

This error has now been corrected in the PDF and HTML versions of the Article.

