# Supplementary material for: Excitation of dark multipolar plasmonic resonances at terahertz frequencies
Source: Sci Rep. 2016 Feb 23;6:22027. doi: 10.1038/srep22027 (PMC4763218; doi:10.1038/srep22027)
Supplement: Supplementary Information [file srep22027-s1.doc]

**Supplementary Information**

**Excitation of dark multipolar plasmonic resonances at terahertz frequencies**

*Lin Chen^a)^, YuMing Wei, XiaoFei Zang, YiMing Zhu* and SongLin Zhuang*

Shanghai Key Lab of Modern Optical System, and Engineering Research Center of Optical Instrument and System(Ministry of Education), University of Shanghai for Science and Technology, No. 516 JunGong Road, Shanghai 200093, China

1. Current address: Oklahoma State University

**Contents**

**Figure S1.** Optical microscope and SEM images of samples. The scales are added in all the images.

**Figure S2.** Dispersion curves of spoof SPPs for one periodic corrugated strips (see inset) with different inner metallic disk radius.

**Figure S3.** Theoretical (top) and experimental (bottom) transmission spectra of the proposed hybrid structure consisted of a corrugated metallic disk and a C shaped resonator with *r*=75 *μ*m.

**Figure S4.** Dispersion curves for the corrugated metallic disk with different *α* (*a/d*). Inset: transmission spectra for corrugated metallic disk coupled to a C shaped dipole resonator with different *α*.

**Figure S5.** Dispersion curves for the corrugated metallic disk with *N*=36 and *N*=60. Inset: transmission spectra for corrugated metallic disk coupled to a C shaped dipole resonator with different *N*.

**Text S6.** Explanation of the dependences of spoof LSPs on the filling ratio *α*(*a/d*) and *N*

**Figure S1. Optical microscope and SEM images of the samples**


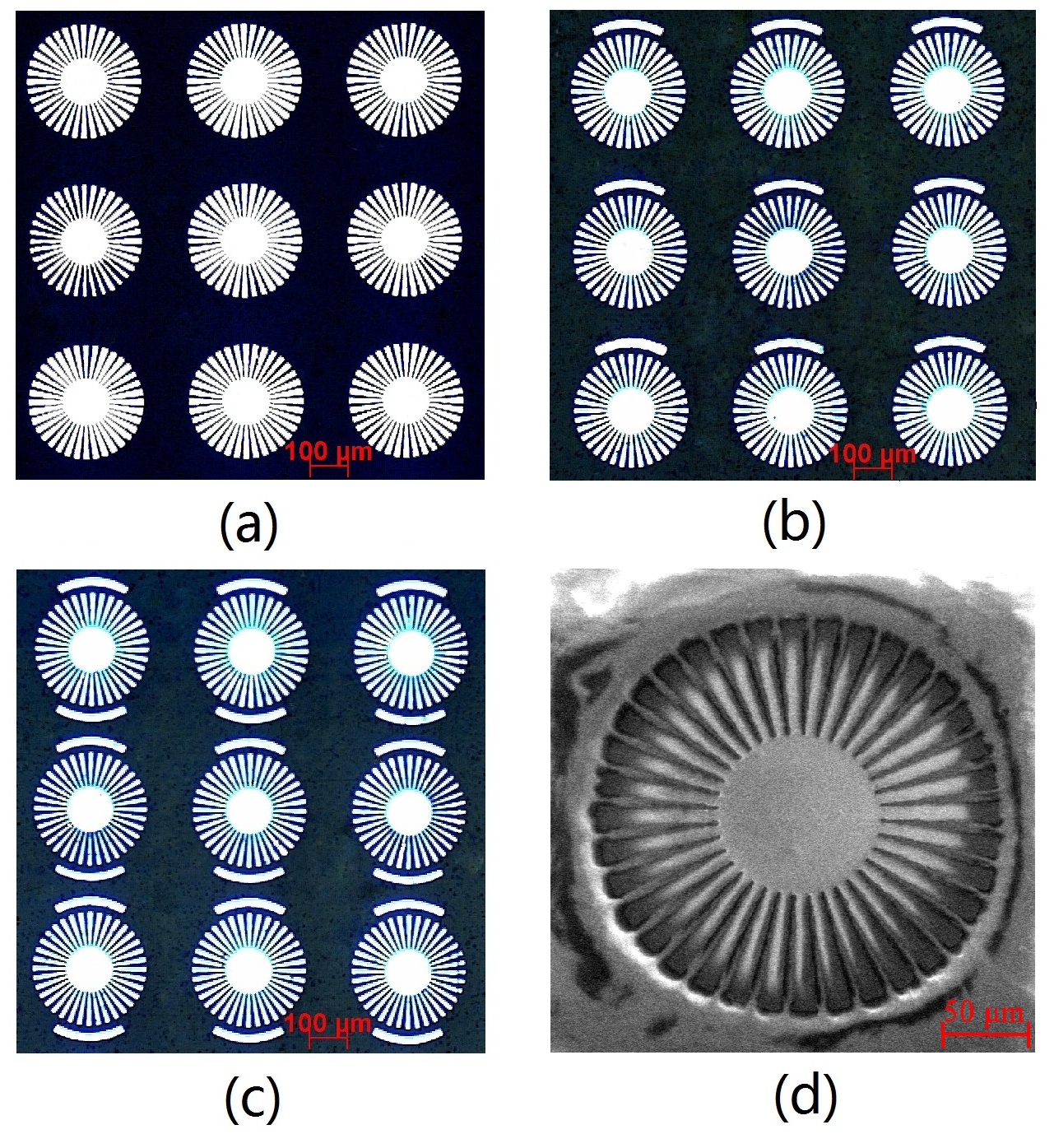


**FIGURE S1.** Optical microscope images of the samples for (a) corrugated metallic disk; (b) corrugated metallic disk coupled to a C shaped dipole resonator; (c) hybrid structure consisting of two identical C shaped resonator placed symmetrically on top and bottom of the corrugated metallic disk. (d) SEM image for the corrugated metallic disk, showing the sectors of the structure. The slight concave-convex area of the SEM image may be due to the strong impact to flexible polyimide substrate by high strength electron beam.

**Figure S2. Dispersion curves of spoof SPPs for one periodic corrugated strips (see inset) with different inner metallic disk radius**


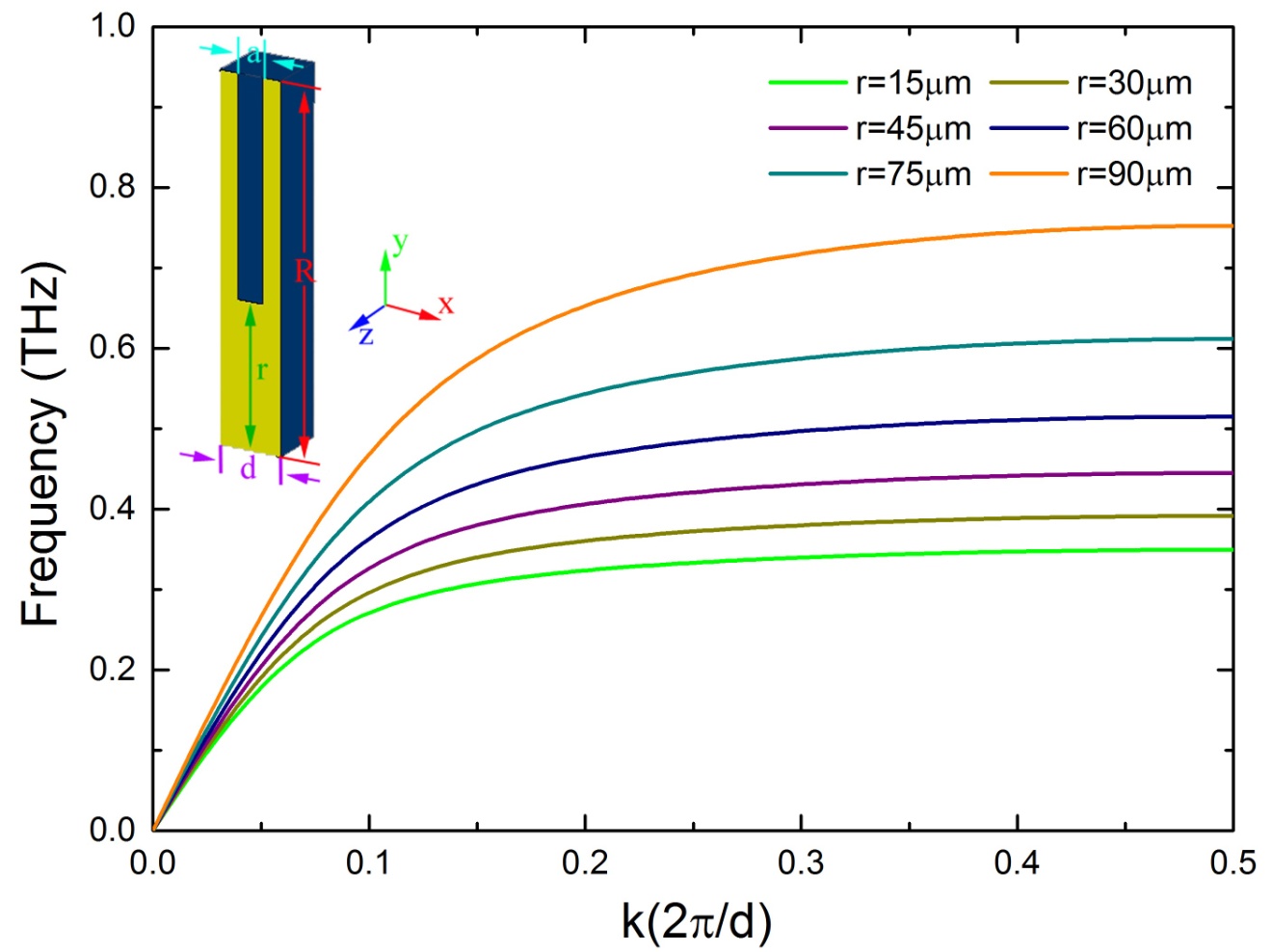


**FIGURE S2.** Simulated dispersion curves of spoof SPPs for one periodic corrugated strips (see inset) with *R* = 150 *μ*m, *N* = 36, *d* = 2π*R*/*N*, *a* = 0.4*d*, and different lengths of *r*.

**Figure S3. Theoretical (top) and experimental (bottom) transmission spectra of the proposed hybrid structure consisted of a corrugated metallic disk and a C shaped resonator with *r*=75*μ*m.**


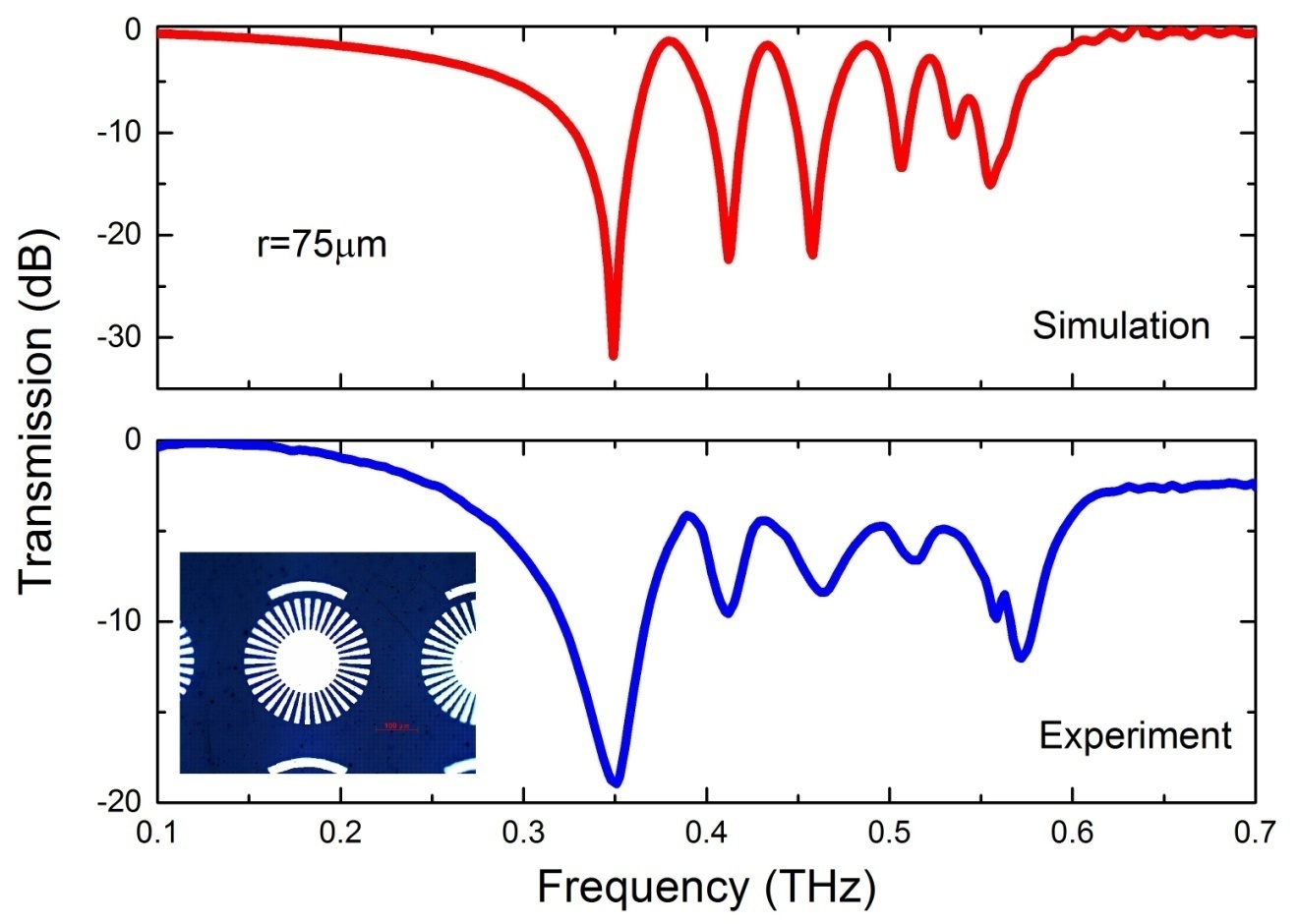


**FIGURE S3.** Theoretical (top) and experimental (bottom) transmission spectra of the proposed hybrid structure consisted of a corrugated metallic disk and a C shaped resonator with r=75*μ*m.(inset) Optical microscope image of the sample. The other parameters are *R* = 150 *μ*m, *N* = 36, *d* = 2π*R*/*N*, and *a* = 0.4*d*.

**Figure S4. Dispersion curves for the corrugated metallic disk with different *α* (*a*/*d*). (inset) Transmission spectra for corrugated metallic disk coupled to a C shaped dipole resonator.**


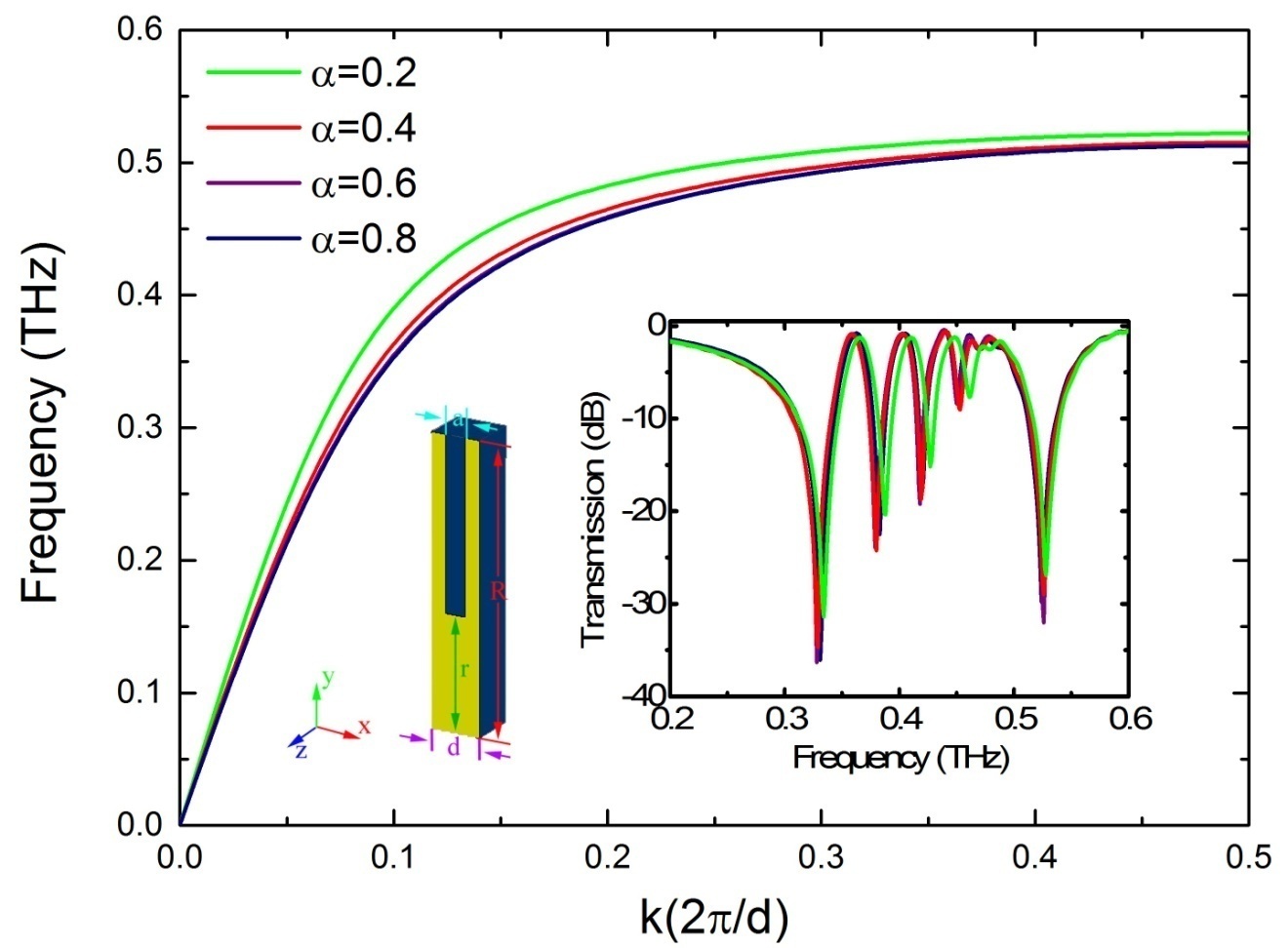


**FIGURE S4.** Dispersion curves for the corrugated metallic disk with different *α* (*a*/*d*). (inset) Transmission spectra for corrugated metallic disk coupled to a C shaped dipole resonator with different *α*. The other parameters are *R* = 150 *μ*m, *N* = 36, *d* = 2π*R*/*N*, and *r*=60*μ*m.

**Figure S5. Dispersion curves for the corrugated metallic disk with different *N*. (inset) Transmission spectra for corrugated metallic disk coupled to a C shaped dipole resonator.**


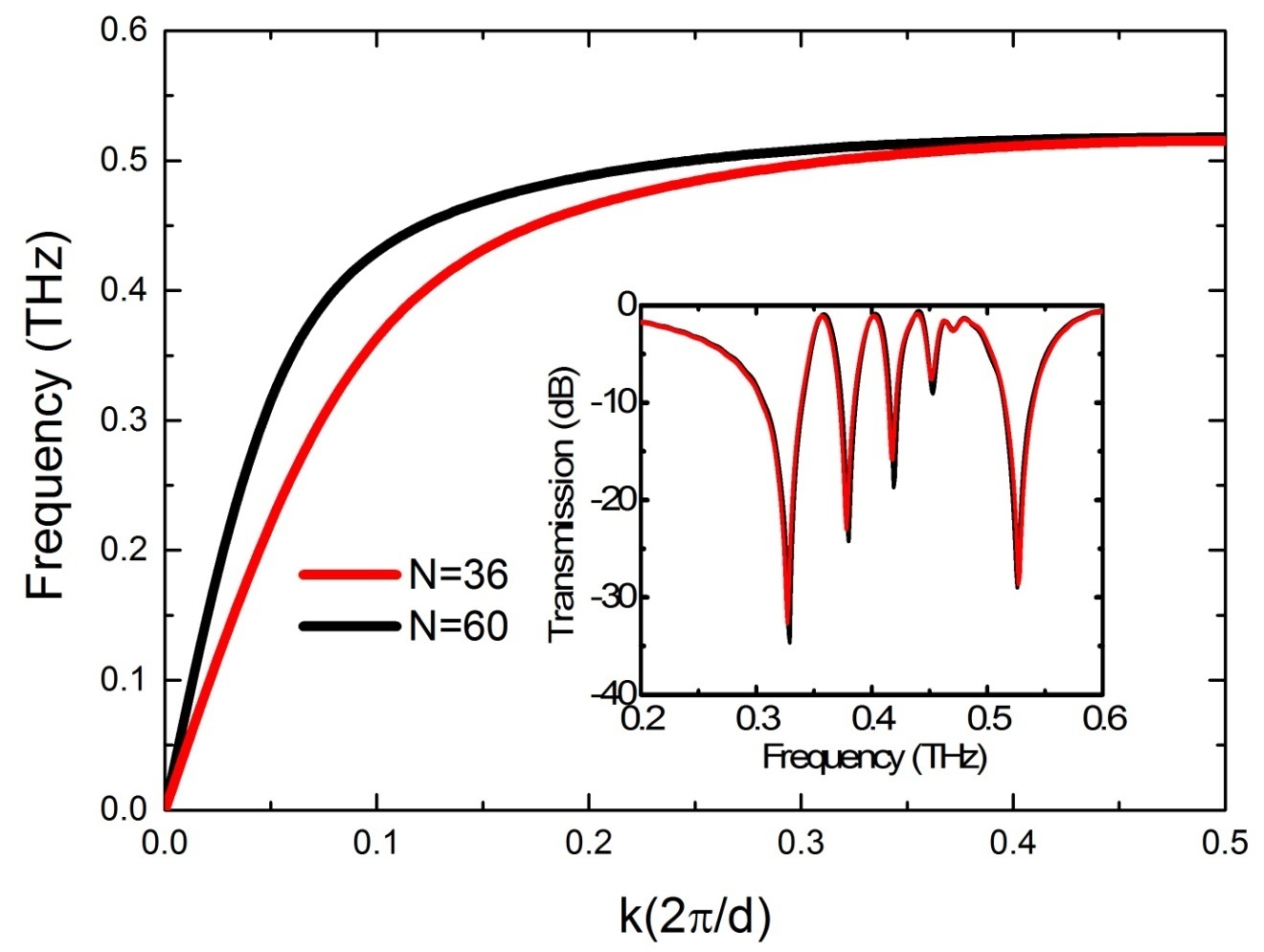


**FIGURE S5.** Dispersion curves for the corrugated metallic disk with different *N*. (inset) Transmission spectra for corrugated metallic disk coupled to a C shaped dipole resonator with *N*=36 and 60. The other parameters are *R* = 150 *μ*m, *α* = *a*/*d =* 0.4, *d* = 2π*R*/*N*, and *r* = 60*μ*m.

**Text S6. Explanation of the dependences of spoof LSPs on the filling ratio *α(a/d)* and *N***

Figure S4 illustrates that the ratio *α* affects slightly the asymptote frequencies of the spoof SPPs, yielding a tiny redshift of the LSP resonances with increasing filling ratio. And the resonance frequencies of the spoof LSPs are independent of the numbers of sectors *N* (in Fig. S5). This observation can be understood through the Equation of asymptote frequency of the spoof surface plasmons, which can be roughly written as *ω_a_* = *πc*/(2*hn_g_*), where *c* is the light speed, *n_g_* is the refractive index of the media filled in the grooves(in this work *n_g_* =1), and *h* represents the depth of the groove, which is *R−r* in Fig. S2. Thus, the asymptote frequency and related spoof LSP resonance frequency are nearly independent of the filling ratio *α* and numbers of sectors *N*.
